# Super-resolution in brain positron emission tomography using a real-time motion capture system

**DOI:** 10.1016/j.neuroimage.2023.120056

**Published:** 2023-03-26

**Authors:** Yanis Chemli, Marc-André Tétrault, Thibault Marin, Marc D. Normandin, Isabelle Bloch, Georges El Fakhri, Jinsong Ouyang, Yoann Petibon

**Affiliations:** a Gordon Center for Medical Imaging, Department of Radiology Massachusetts General Hospital, Harvard Medical School, Boston, MA, United States; b Department of Computer Engineering, Universté de Sherbrooke, Sherbrooke, QC, Canada; c Sorbonne Université, CNRS, LIP6, Paris, France; d LTCI, Télécom Paris, Institut Polytechnique de Paris, France

**Keywords:** Super-resolution, PET/CT, 3D brain PET imaging, Real time, Tracking, Reconstruction

## Abstract

Super-resolution (SR) is a methodology that seeks to improve image resolution by exploiting the increased spatial sampling information obtained from multiple acquisitions of the same target with accurately known sub-resolution shifts. This work aims to develop and evaluate an SR estimation framework for brain positron emission tomography (PET), taking advantage of a high-resolution infra-red tracking camera to measure shifts precisely and continuously. Moving phantoms and non-human primate (NHP) experiments were performed on a GE Discovery MI PET/CT scanner (GE Healthcare) using an NDI Polaris Vega (Northern Digital Inc), an external optical motion tracking device. To enable SR, a robust temporal and spatial calibration of the two devices was developed as well as a list-mode Ordered Subset Expectation Maximization PET reconstruction algorithm, incorporating the high-resolution tracking data from the Polaris Vega to correct motion for measured line of responses on an event-by-event basis. For both phantoms and NHP studies, the SR reconstruction method yielded PET images with visibly increased spatial resolution compared to standard static acquisitions, allowing improved visualization of small structures. Quantitative analysis in terms of SSIM, CNR and line profiles were conducted and validated our observations. The results demonstrate that SR can be achieved in brain PET by measuring target motion in real-time using a high-resolution infrared tracking camera.

## 1. Introduction

For the last several decades, Positron Emission Tomography (PET) neuroimaging has provided unique insights into brain function, including quantitative measurements of cerebral glucose metabolism, blood flow as well as numerous receptors, transporters and enzymes (Gunn et al., 2015). More recently, the advent of radiotracers binding to misfolded proteins such as amyloid and neurofibrillary tangles (tau), has ushered in a new era in PET imaging of neurodegenerative diseases, accompanied with new requirements in terms of image quantification and processing. In particular, imaging of tau pathology, especially in early disease stages, is fueling a need for improved PET spatial resolution (Lecomte et al., 2022) to allow for accurate imaging of more focal tracer uptake patterns and small brain structures (e.g., entorhinal cortex) (Schöll et al., 2016).

The effective spatial resolution of PET images is limited by several factors. One relates to the physical effects of positron emission and annihilation, such as the positron range and photon non-collinearity. The other pertains to limitations of hardware and instrumentation including block effects and the width of the crystals, the latter being by far the main resolution-limiting factor in clinical PET scanners (Li et al., 2014). Another important effect limiting PET resolution is the movement of the subject during the acquisition. Indeed, a brain scan typically extends over several minutes up to hours for some dynamic acquisitions and any substantial head motion during the scan will introduce blurring in the final reconstructed image.

The PET spatial resolution could be enhanced by improving detector designs (e.g., reducing crystal width) and electronics; however, these modifications would incur additional costs and would be difficult to implement in existing PET scanners. In this paper, we show that it is possible to enhance the spatial resolution of brain PET images in real time by harnessing the usually undesired head motion using an image processing technique known as super-resolution (SR) (Peleg et al., 1987).

**Fig. 1.**
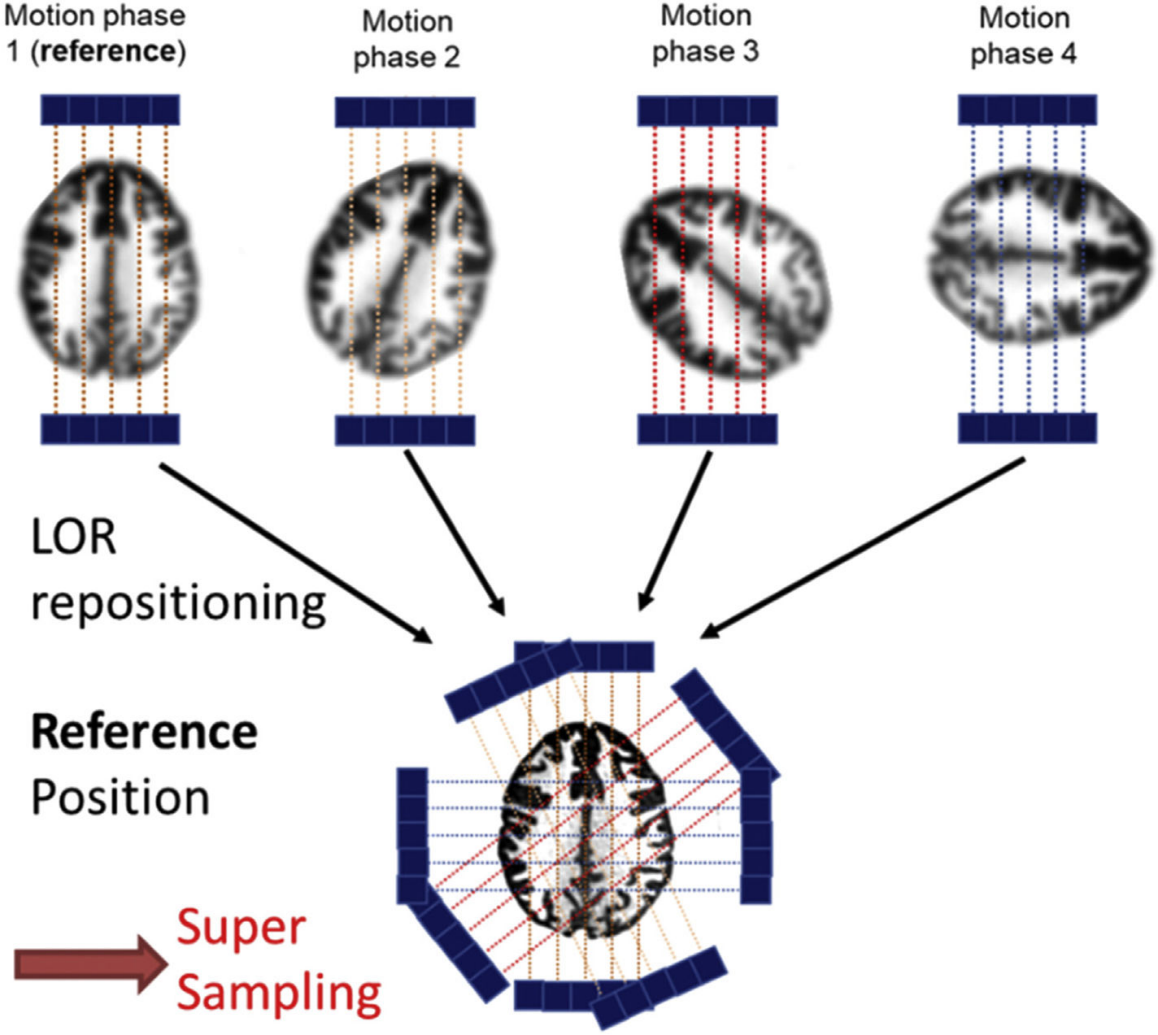
Principle of super-resolution in brain PET. The object is sampled in different positions due to motion. To achieve SR, motion transformations measured by a high-resolution optical tracking are applied to the acquired LORs during image reconstruction to estimate a higher resolution PET image in a common oversampled reference grid.

Super-resolution methods seek to generate a high-resolution (HR) image from a single or multiple low-resolution (LR) images acquired in different positions. The basic idea of SR is to combine the partial non-redundant spatial sampling information contained in the LR images to produce the HR image. The partial non-redundant information contained in these LR images is generally obtained by introducing subpixel shifts to the system through uncontrolled or controlled - but accurately known- movement between the imaging device and the object (Barreto et al., 2006). SR estimation then consists in reversing this shifting process by aligning the LR observations to subpixel accuracy and combining them into an HR image grid (Peleg et al., 1987). In PET imaging, the partial non redundant information can potentially be obtained by moving the scanned object inside the field of view of the scanner and by correcting the position of the line of responses (LOR) at a subpixel accuracy to a common reference (Fig. 1).

Different methods have been proposed over the years to achieve SR in PET imaging. Dagher and Thompson’s work consisted in a hardware-based approach where the scanner detector rings were physically moved with high precision following a precession or “wobbling” movement. By doing so along a specific path, their work lead to oversampled projections (Dagher and Thompson, 1985). Wernick and Chen showed that further resolution enhancement can be obtained by applying the same hardware-based super-resolution approach, utilizing detector motion, to directly improve the quality of the projection matrix before tomographic reconstruction (Wernick and Chen, 1992). In their work, a filtered back-projection using the super sampled projection matrix was applied on the data yielding substantially improved images in contrast and SNR. Later, instead of moving the detectors, Kennedy et al. created multiple low-resolution frames by shifting and rotating the scanned object. For a precise control of the motion, they used a special jig comprised of a translational stage and a micrometer. They showed an increase in both resolution and contrast on a clinical scanner (Kennedy et al., 2006). Instead of reconstructing the separately constructed high-resolution (HR) sinograms or applying SR to already reconstructed low-resolution images, more recent work integrated the SR estimation directly into the iterative process of the Maximum Likelihood Expectation Maximization (MLEM) image estimation. In Li et al. (2014) the authors present a super-sampling data acquisition model based on the physical processes of PET acquisition including, the forward model, blurring, downsampling, and motion as its building blocks. Based on their model, they used and extended MLEM algorithms to reconstruct images directly from oversampled data sets leading to improved resolution, contrast, and signal to noise ratio (SNR). In Verhaeghe and Reader, (2010), a SR PET data framework was introduced to generalize the image reconstruction process in the presence of any type of acquisition motion. The authors studied and exploited wobble or random motion to increase resolution. However, they discretized motion, underexploiting the benefits of continuous motion. With the recent advances in Machine Learning, PET super-resolution using deep learning has emerged in the recent years. Song et al. used a convolutional neural network to achieve super resolution on a single acquisition without making use of object motion. Instead, they incorporated high resolution (HR) anatomical information based on magnetic resonance (MR) imaging as well as spatial location information to model the spatially varying blur inherent to PET images, inducing visually finer structural details (Song et al., 2020).

However, to the best of our knowledge, all the mentioned methods still rely on having multiple fixed positions of the scanned object (step and shoot fashion) or end up discretizing and binning continuous movement (Verhaeghe and Reader, 2010). As mentioned by the authors in (Song et al., 2020), CNN-based SR main limitation is that it relies on supervised learning and therefore, requires paired low resolution and high-resolution PET images for training. It is still an open problem to find a way to achieve SR in an actual clinical setup using commercial scanners and in scenarios where a patient can be subject to small uncontrolled movements. The purpose of this work is to show that SR can be achieved with a state-of-the-art high-resolution optical tracking device that is used to measure continuously, in real-time, the undesired random head movement with a very high spatial and temporal accuracy during a PET acquisition, achieving SR using a commercial clinical scanner. The main contributions of this work are the robust temporal synchronization, the accurate spatial calibration between the optical tracking device and a PET/CT scanner and the use of the real time motion information into a list-mode based reconstruction scheme.

## 2. Materials and methods

### 2.1. Code and data availability statement

The data used in this work will be made available upon reasonable request, contingent on the obtention of a formal data sharing agreement with the institution(s) owning such data. However, the code in its current development is confidential. It will be made available in the future as part of an open-sourced reconstruction engine project for PET imaging or executable binary files will be provided upon reasonable request.

### 2.2. Overview

Our objective is to harness the usually undesired head motion that typically degrades PET spatial resolution to actually enhance it using SR. To accomplish that, we need to measure the unpredictable and potentially continuous head motion occurring during a PET scan with high spatial and temporal resolution.

Standard motion tracking techniques, which typically rely on registration of reconstructed PET images, have limited temporal and spatial resolution, and often fail in presence of rapid or continuous head motion. An alternative approach is to use external optical tracking systems (Rahmim et al., 2007), which usually provide motion information with excellent temporal and spatial resolutions.

### 2.3. High-resolution motion tracking device

In this work, we use a high-resolution optical tracking device along with a state-of-the-art clinical PET/CT scanner, the GE Discovery MI (GE Healthcare, United States), to achieve SR in brain PET studies.

Several 3D external motion tracking systems have been employed for motion correction of brain PET (Picard and Thompson, 1997), (Fulton et al., 2002). Here, we use the Polaris Vega, a tracking system manufactured by Northern Digital Inc. (NDI, Canada). The Polaris Vega is a device approved for medical environments that tracks a “tool” or “target” where spherical reflective markers are mounted in a specific geometry recognized by the tracker. It can track a target with a 0.12 mm volumetric accuracy. The motion capture frame rate can be selected from 20, 30, to 60 Hz. Temporal synchronization with external devices is only supported through the IEEE 1588 PTP (Precise time Protocol) standard. However, the GE Discovery MI cannot directly be interfaced in this manner, thus requiring an alternate method to align the system with the PET scanner time base. Accurate spatial calibration of both devices is required as one must relate the tracker and scanner coordinate frames.

### 2.4. Temporal synchronization, spatial calibration between the Polaris Vega and the PET/CT scanner and integration of the motion in PET reconstruction

There are three key steps to achieve our SR: the first is the development of a communication interface between the Polaris Vega camera and the scanner to temporally synchronize the two devices. The second step is the accurate spatial calibration between the apparatuses. The last step consists in integrating the motion information from the Vega in a list-mode event by event motion-compensated PET reconstruction framework to achieve SR. Those steps are explained in the following sections. We tested and validated the developed method with three different experiments: one using a mini hot spot phantom (Data Spectrum) for validation, another using a brain Hoffman phantom, and an in vivo study with a non-human primate.

#### 2.4.1. Temporal synchronization

To achieve temporal alignment between the tracking device and the PET scanner, the Raspberry Pi4 (Rpi) platform was used as a host to control PTP synchronization and to generate pulses to be injected into the scanner gating signal input and incorporated into the PET listmode data stream. The PTP is a protocol used to synchronize clocks throughout a computer network. It achieves clock accuracy in the sub-microsecond range on a local area network, making it suitable for measurement and control systems (Eidson, 2006). Thanks to this tool, no specific time delay correction implementation is needed (Spangler-Bickell et al., 2016). The Raspberry Pi4 first achieves synchronization with the Vega by providing a PTP master reference. Since the Vega provides a PTP timestamp with every frame, the Raspberry Pi4 can then determine when to generate a pulse synchronized with motion frame capture.

**Fig. 2.**
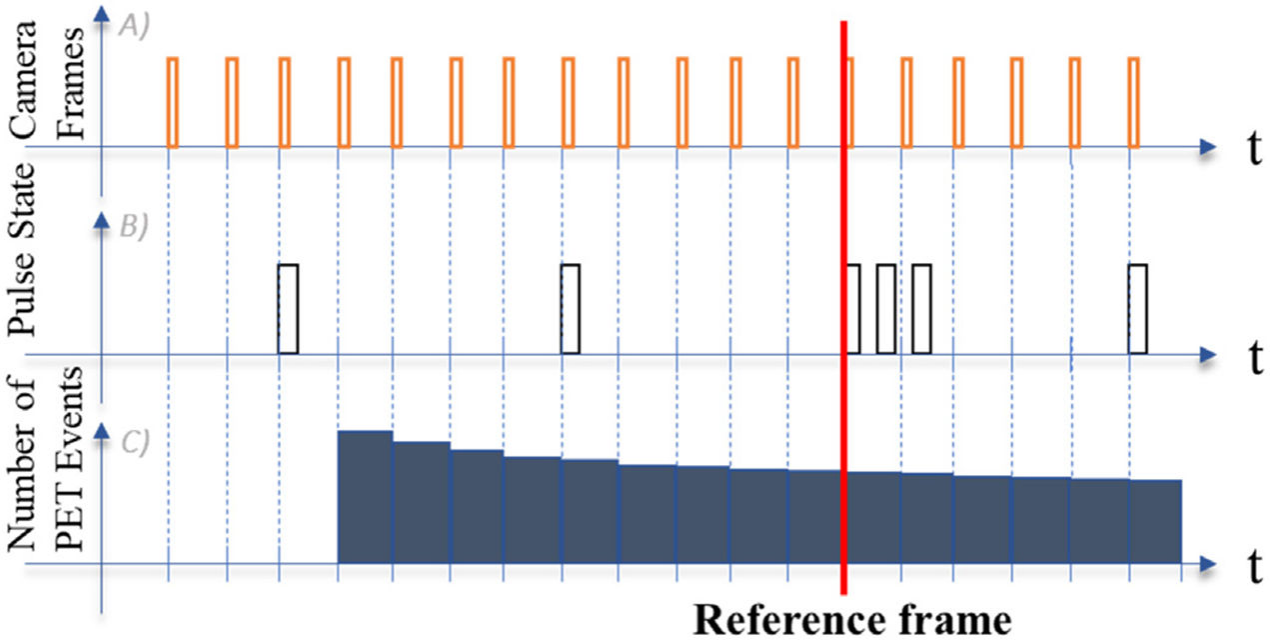
Temporal synchronization between the Polaris Vega and the PET/CT scanner. A) Camera frame timestamps are sent in the RPi. B) Aligned RPi timestamps are generated and sent to the listmode data stream, and a reference frame is defined by a specific pattern of pulses C) PET events are aligned with the camera stamps post acquisition.

After the PET acquisition starts, a pattern of pulses is sent to the gating interface to define the starting frame time and position. In addition, pulses are sent periodically to the list mode data stream to ensure that no time drift occurs between the PET system and the PTP master counter (Fig. 2).

#### 2.4.2. Spatial calibration

Since the PET and Polaris Vega coordinate systems are not intrinsically aligned, a transformation matrix must be determined to convert the recorded motion tracking data from the Polaris Vega into the PET coordinates. A standard solution is to use a radioactive point source that is rigidly placed at the origin of a tracked target, and simultaneously scan and measure them at various positions within the PET scanner and tracker fields of view. It is then possible to determine a suitable transformation between the coordinate systems by finding the relationship between the two sets of coordinates (Fulton et al., 2002). However, such an approach could potentially limit resolution recovery gain of SR since the accuracy of the spatial calibration matrix would be close to that of the intrinsic PET resolution. For SR, the accuracy of the spatial calibration should be higher than the intrinsic PET resolution. Here, rather than relying on PET point sources, we determined the calibration matrix using the CT, which shares the same image space as the PET component but has a much higher spatial resolution.

The spatial alignment transformation between the Polaris Vega and the scanner coordinate spaces is determined by six paired measurements of high-resolution CT scans (0.7 × 0.7 × 0.6 mm^3^) and Polaris tracking of individual reflective markers. The markers positions are chosen regularly spaced across the field of view of the CT. In the Polaris camera space, the different positions of the center of a marker (which is a sphere) are directly given by the camera. The corresponding positions of the markers in the CT space are manually spotted in the acquired 3D volumes. A matrix $M_{c}$ representing the 3-D rigid transformation between the two coordinate systems (PET scanner and Polaris camera coordinate frames) is found by measuring the position of the same set of points in both spaces simultaneously. Using the two sets of corresponding 3-D point data, the optimal solution for $M_{c}$ in terms of least square optimization is found using singular value decomposition (SVD) on the covariance matrix built from the sets of points (Arun et al., 1987). Using high-resolution CT rather than multiple tracked PET point sources ensures that the accuracy of the measured transformations is greater than the PET intrinsic spatial resolution, which is required to achieved super-resolution.

To apply the spatial alignment in subsequent experiments in which the relative position of the Polaris and scanner may have changed, we use a reference target built with marker rigidly affixed to the gantry (Fig. 4). The camera simultaneously tracks the position of the mobile target and the reference target relative to the Polaris reference frame. This allows positioning the camera anywhere for each experiment without having to recalibrate the system.

The global transformation matrix L allowing to express the position of the tracked marker from the reference camera space to the PET image space, is given by:

(1) $$L=M_{\;c}M_{\;RefT}M_{\;T}^{\prime -1}\;M_{\;c}^{-1}$$

where $M_{c}$ is the aforementioned calibration matrix relating the PET image space and Polaris Vega coordinate space, $M_{\mathrm{RefT}}$ is the reference rigid transformation matrix that represents the position where all the other positions are registered to, and $M_{T}$ is the current rigid transformation matrix given by the Polaris Vega at each time point.

**Fig. 3.**
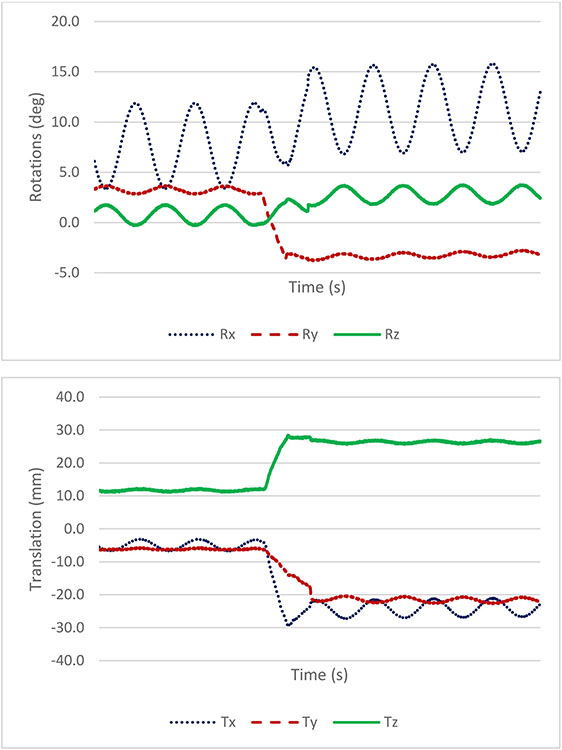
Examples of motion transformations measured by the Polaris Vega camera in the PET image space for a phantom experiment with continuous movement. Rx, Ry and Rz are the rotation angles and Tx, Ty, Tz are the translations of the tracked target in the image space spanned by x, y, and z axes.

Using this set of transformations with our calibration method, we can relate the coordinates of the tracked target from the camera space to the PET image space with high accuracy (see the example in Fig. 3).

#### 2.4.3. List-mode based ordered subset expectation maximization (OSEM) motion corrected reconstruction for SR

For a given scan, the motion data obtained by the Polaris is used to transform the endpoints of measured LORs to a common reference frame on an event-by-event basis at the corresponding time. Let $L_{t}$ model the effect of motion at time frame t (i.e., rigid-body transformation from the reference frame to t) in the LOR space and $i_{m}$ represent LOR i∈[1…I] associated with list-mode event m detected during frame t; thus we have $i_{m}^{\prime }=L_{t}^{-1}(i_{m})$ where $i_{m}^{\prime }$ denotes the transformed LOR for event m after motion correction. To achieve SR, a list-mode OSEM reconstruction algorithm with LOR-by-LOR motion compensation was implemented using the formulation in Spangler-Bickell et al. (2019). Derived from a classic list-mode, this formula integrates motion information directly into the system matrix P:

(2) $${\hat{\rho }}_{SR_{j}}^{l+1}=\frac{{\hat{\rho }}_{SR_{j}}^{l}}{\tilde{S_{j}}}\sum_{m=1}^{M}P_{i_{\;m}^{\prime },j}\frac{1∕I_{i_{\;m}^{\prime }}}{\sum_{k=1}^{J}P_{i_{\;m}^{\prime },k}\;{\hat{\rho }}_{SR_{k}}^{it}+\frac{S_{i_{m}}+R_{i_{m}}}{a_{i_{m}}N_{i_{m}}}}$$

where ${\hat{\rho }}_{SR_{j}}^{it}$ is the SR image value at voxel j∈[1…J] and iteration l in the reference frame, M is the total number of events in the list-mode file, $I_{i_{m}^{\prime }}$ is the attenuation correction factor for attenuating material undergoing motion (e.g., head) for the transformed LOR $i_{m}^{\prime }$, $S_{i_{m}}$ and $R_{i_{m}}$ are respectively the estimated scatter and random contributions, $a_{i_{m}}$ is the combined moving (e.g., head) and non-moving (e.g., scanner bed) attenuation correction factor for uncorrected LOR $i_{m}$, $N_{i_{m}}$ is the detector sensitivity for LOR $i_{m}$.

$P_{i,j}$ is an element of the PET system matrix defined as:

(3) $$P_{i,j}=\sum_{l=1}^{I}B_{proj_{i,l}}\sum_{k=1}^{J}G_{l,k}B_{img_{k,j}}$$

where $G_{i,j}$ accounts for the geometric probability that an event generated in voxel j is detected along LOR i; $B_{img_{k,j}}$ and $B_{proj_{i,l}}$ are elements of PSF kernel matrices in the image space and in the projection space, respectively. The kernel values for each component were given by the scanner manufacturer. PSF effects are accounted for in two steps during reconstruction: the spatially invariant 3D Gaussian smoothing kernel $B_{\mathrm{img}}$ is first applied to the image at each update and then LORs are spread in the projection step according to the spatially varying PSF kernel $B_{\mathrm{proj}}$, and vice-versa during back-projection. The image-space component models the positron range and allows using slightly narrower PSF kernels in the projection space, which speeds up the projection and back-projection operations during each update. Integrating smoothing also makes the reconstruction more robust to slight high-frequency artifacts that might appear during the iterations (Deller et al., 2021).

$\tilde{S_{j}}$ is the time-averaged sensitivity image value at voxel j that accounts for LOR normalization factors and hardware attenuation. It is defined as (Spangler-Bickell et al., 2019):

(4) $$\tilde{S_{j}}=\frac{1}{T}\sum_{t}\sum_{j=1\ldots J}L_{t,j\to j^{’}}\sum_{i=1\ldots I}P_{ij}H_{i}N_{i}$$

where T is the total number of motion frames $H_{i}$ is the attenuation correction factor for non-moving components for LOR i.

$L_{t}$ being measured at a very high resolution, we can increase spatial sampling by providing complementary information which can be exploited by the OSEM algorithm (Fig. 1) to reconstruct a PET image on a finer voxel grid.

### 2.5. Phantom and non-human primate experiments

Two phantoms, a Mini Hot Spot and a Hoffman, respectively filled with 74 MBq and 111 MBq of 18F, were scanned for 15 min in list-mode on the PET/CT scanner while undergoing continuous rotation/translation movements introduced by a QUASAR system (Modus QA). Similarly, an anesthetized male rhesus monkey administered with 407 MBq 18F-FDG was scanned for 15-min (60 min after tracer injection) in list-mode with continuous and random head motion induced manually. This experiment complied with the ARRIVE guidelines and was carried out in accordance with the National Institutes of Health guide for the care and use of Laboratory animals (NIH Publications No. 8023, revised 1978).

**Fig. 4.**
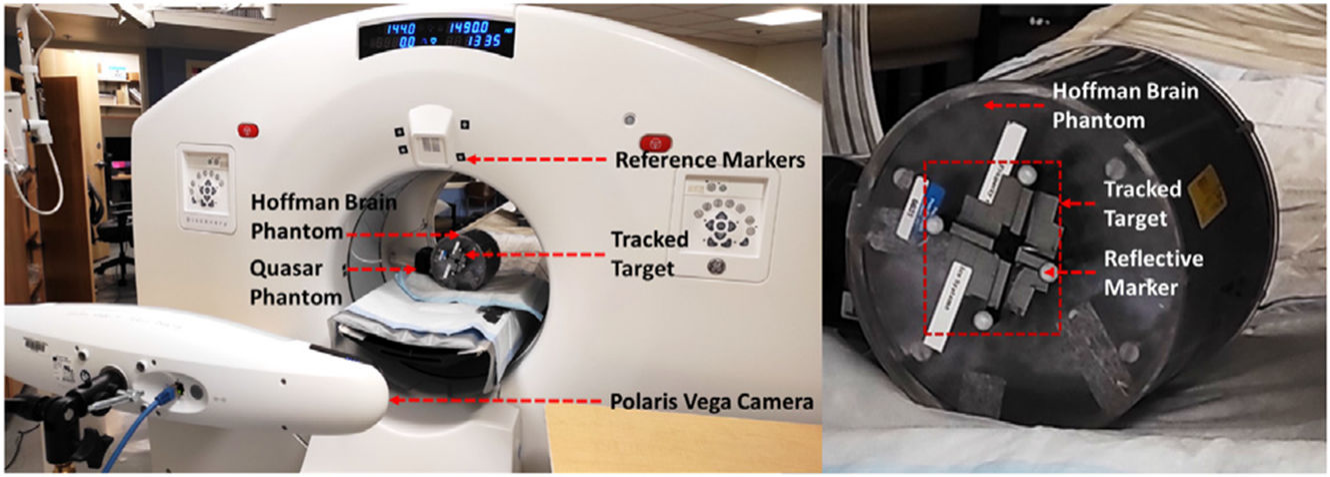
Experimental setup used for the Hoffman phantom experiment showing the placement of Polaris Vega relative to the GE DMI scanner, the Hoffman phantom on which is attached the tracked markers, the Quasar phantom, and the reference markers.

For all the scans, motion was tracked at all times using the Polaris and reflective markers rigidly attached to the targets (see Fig. 4 for the Hoffman phantom study). For the NHP, markers were rigidly attached to the skull using an adhesive bandage. For each study, reference static PET acquisitions were also performed without inducing movement. Listmode data were reconstructed with three different methods: (A) OSEM algorithm with PSF modeling applied to the static reference scan data (2 mm voxel size), (B) OSEM with PSF modeling applied to the static scan on smaller voxel size (0.8 mm for the mini hot spot and 1 mm for the Hoffman), (C) proposed SR algorithm applied to the moving scan with 0.8 or 1 mm voxel size. The iteration numbers were chosen to match image noise levels for all methods.

### 2.6. Evaluation of image quality

We evaluate the quality of the super-resolved PET images via two conventional measures in image processing, namely the Contrast-to-Noise Ratio (CNR) and the Structural Similarity Index (SSIM) (Wang et al., 2004). The CNR gives a contrast index between different regions, in the phantoms or NHP, relative to the noise level. Here, it was computed as:

(5) $$CNR=\frac{\mu Target\;-\;\mu Background}{\sigma \text{Background}}$$

μTarget is the mean value in active target regions, μBackground is the mean value in the background, and σBackground is the standard deviation in background, modeling the noise level. In those studies, we chose the background in a large region with relatively uniform activity distribution for both phantoms and non-human-primate.

The SSIM evaluates the structural similarity between two images (e.g., ground truth or reference image vs. estimated image). It combines three terms: luminance, contrast features, as well as an image correlation term. For both the mini hot spot and Hoffman phantoms, CT provides high-resolution reference images for SSIM calculation. Hence, it was computed as:

(6) $$\text{SSIM}(\text{PET,CT})=\frac{\left(2\mu PET\mu CT+C1)(2\sigma PETCT+C2\right)}{\left(\mu 2\text{PET}+\mu 2CT+C1)(\sigma 2PET+\sigma 2CT+C2\right)}$$

where μCT is the mean CT value in the ROI, μPET is the mean PET value in the ROI, σCT is the standard deviation of CT values in the ROI, $\sigma_{\text{PET}}$ is the standard deviation of PET values the ROI, σPETCT is the cross-covariance for PET and CT in the ROI and $C_{1}$ and $C_{2}$ are regularization constants that helps avoiding instability for image regions where the local mean or standard deviation is close to zero.

**Fig. 5.**
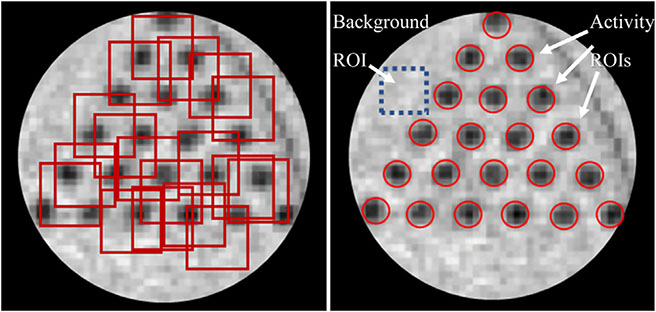
Illustration of ROIs selection for SSIM and CNR calculation in the Mini Hot Spot phantom. Left: Examples of randomly selected ROIs in a window drawn around the 2.4 mm rods in the reference CT image. Right: Selection of ROIs for CNR calculation. The dashed blue square shows the background ROI.

The SSIM was only calculated for the two phantoms which had a CT reference available. The regions of interest were randomly selected across multiple slices on windows centered in regions where structures are small, while the CNR was calculated by selecting multiple regions with activity and a unique background region (Fig. 5).

Line profiles were used to further quantitively assess resolution recovery from the SR method. Those were drawn across small structures typically at the limit of the scanner resolution capabilities.

## 3. Results

### 3.1. Phantom studies

#### 3.1.1. Mini hot spot phantom

**Fig. 6.**
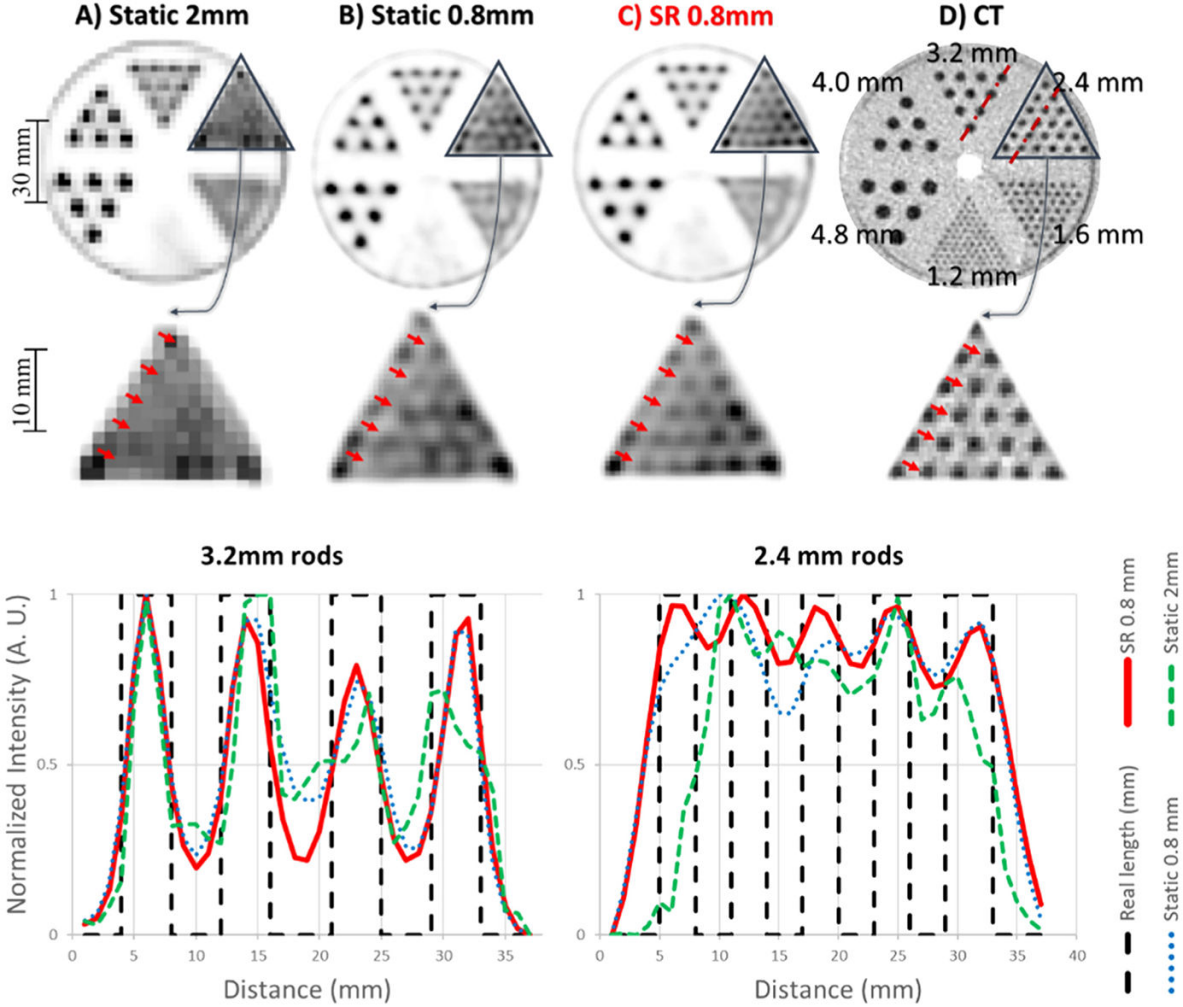
Results of the Mini Hot Spot phantom study. Top row: same PET slice reconstructed with A) static OSEM-PSF with 2 mm voxels, B) static OSEM-PSF with 0.8 mm voxels, C) proposed SR method with 0.8 mm voxels, and D) corresponding CT slice (note that the CT image can be treated as a high-resolution reference image for the experiment). Bottom row: Corresponding line profiles for the different methods.

The mini hot spots phantom was moved with a range of approximately ±20° and ±40 mm, with motion along all 6 degrees of freedom (see sample in Fig. 3). The data set consisted of about 1 billion events spanning 15 min for both the static and moving acquisitions. Fig. 6 shows the results of the listmode based reconstructions of the static data for the standard 2 × 2 × 2.8 mm^3^ voxel size as well as in 0.8 × 0.8 × 2.8 mm^3^ voxel size (that we refer to as static 2 mm and static 0.8 mm, respectively). The data from the moving acquisition were reconstructed with voxel size of 0.8 × 0.8 × 2.8 mm^3^ to generate SR images (referred to as SR 0.8 mm). Fig. 6 also shows the aligned CT with voxel size of 0.7 × 0.7 × 0.625 mm^3^ as the reference.

The line profiles passing through 3.2 and 2.4 mm rods across all these reconstructions are shown in the bottom panel of Fig. 6. Note that the reference frame for SR reconstruction was that of the static data. Therefore, the SR reconstruction is well aligned with the static reconstruction without needing additional image registration. Hence, for clinical studies, the PET data can be corrected to the reference frame of the attenuation map to ensure that these are well aligned.

As shown visually and by the line profile in Fig. 6, the 3.2 mm rods of the static and SR reconstructions are all resolved correctly. This is expected as the intrinsic resolution of the scanner allows resolving structures of this size. However, the 2.4 mm rods cannot be resolved in both static reconstructions, whereas they can clearly be visualized with the SR reconstruction, indicating an improvement in spatial resolution.

#### 3.1.2. Hoffman phantom

Similar results were obtained using the Hoffman brain phantom, which was moved in a similar manner as the mini hot spot phantom albeit with more movement amplitude along the axial direction of the scanner.

**Fig. 7.**
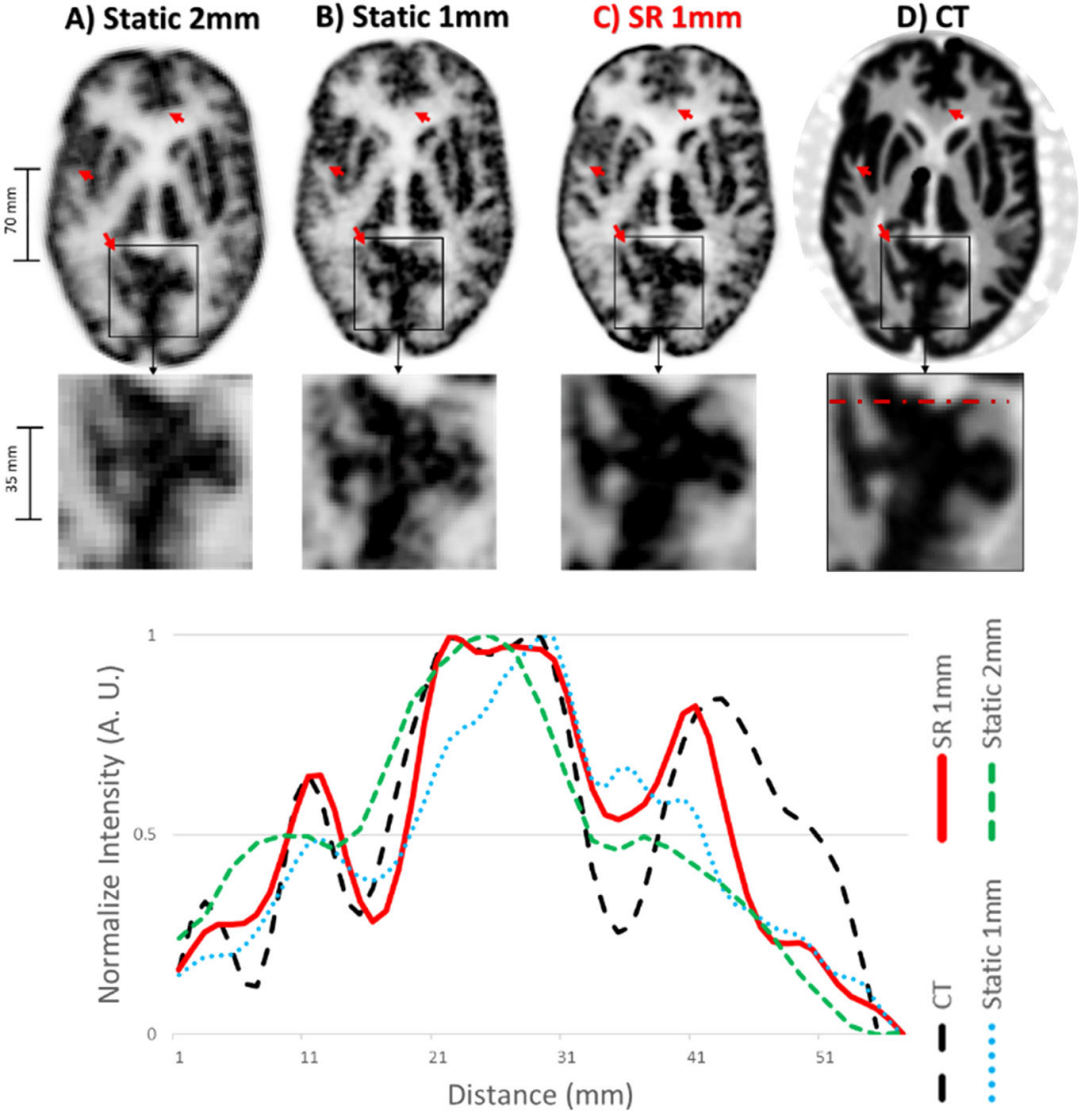
Result of the Hoffman phantom study. Top panel: same PET slice reconstructed with A) static OSEM-PSF with 2 mm voxels, B) static OSEM-PSF with 1 mm voxels, C) proposed SR method with 1 mm voxels, and D) corresponding CT slice (the CT image can be considered as a high-resolution reference). Bottom panel: corresponding line profiles.

The reconstructed list-mode data for each method and a line profile passing through small structures of the brain across all these reconstructions are shown in Fig. 7.

The proposed SR reconstruction method yielded PET images with visibly improved spatial resolution compared to standard and static reconstructions with the same 1 × 1 × 1 mm^3^ voxel size (here referred to as static or SR 1 mm), allowing for a better characterization of small cortical and subcortical brain phantom structures (see Fig. 7). Line profiles confirmed the improvement in spatial resolution for the SR image as well as an improved correspondence with high-resolution CT as compared to the conventional methods.

### 3.2. In vivo study

The rhesus monkey administered with 11 mCi 18F-FDG was sedated and scanned for 15-min in list-mode without motion, followed by a 15 min acquisition with continuous head motion induced manually. The same reconstruction parameters as in the Hoffman Phantom experiment were used with a voxel size of 1 × 1 × 1 mm for SR.

**Fig. 8.**
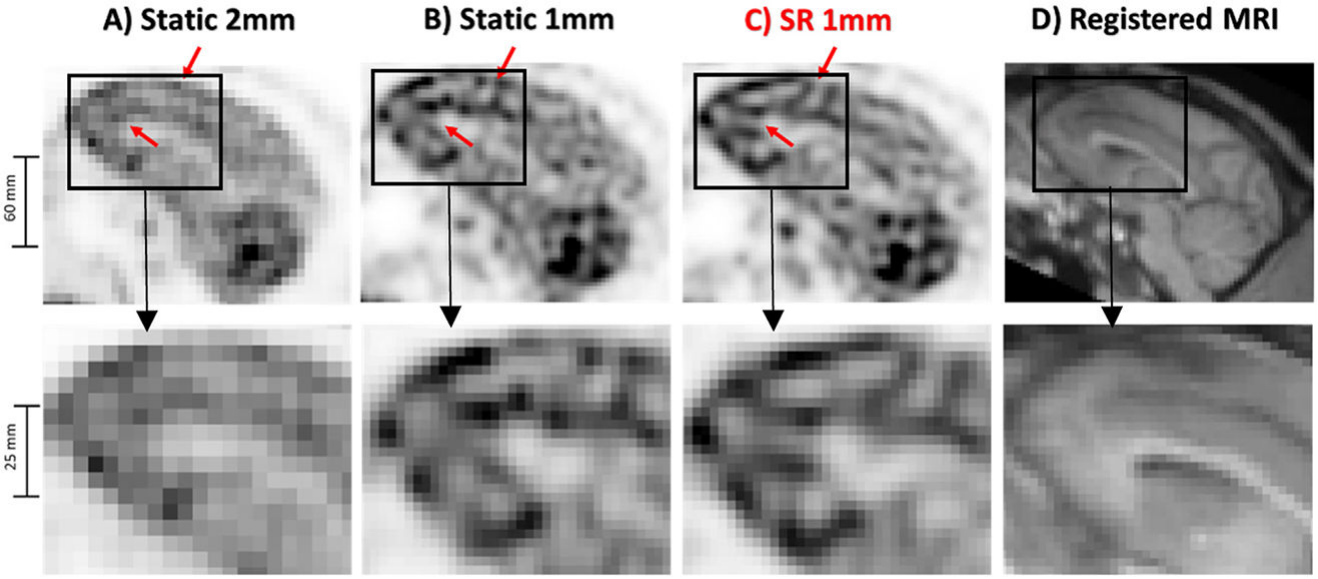
Results of the 18F-FDG NHP in vivo study. Same PET slice reconstructed with A) static OSEM with 2 mm voxels, B) static OSEM with 1 mm voxels, C) proposed SR method with 1 mm voxels, and D) corresponding MR slice.

Fig. 8 shows a sagittal slice through the brain for the NHP study. Specific brain regions, such as the frontal lobe, can be better resolved after SR.

### 3.3. Quantitative analysis

**Fig. 9.**
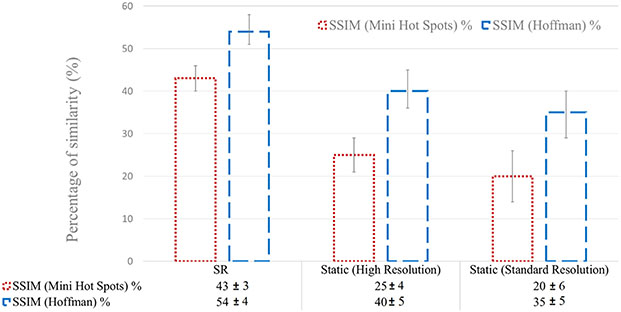
SSIM results for the Mini Hot Spots Phantom (dotted red) and the Hoffman Phantom (dashed blue). The bar plot shows the mean SSIM as described in Fig. 6. Error bars representing the standard deviation are also plotted.

**Fig. 10.**
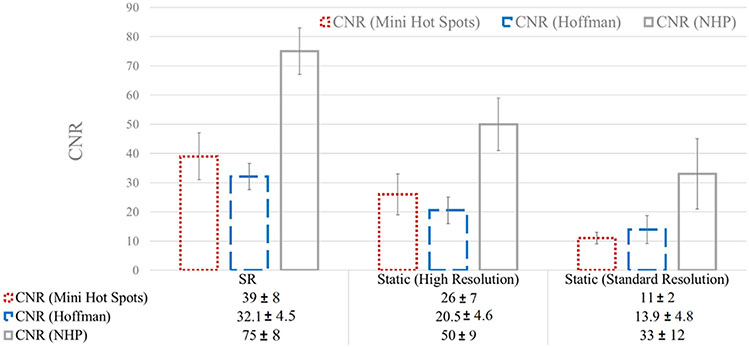
CNR results for the Mini Hot Spots Phantom (dotted red), the Hoffman Phantom (dashed blue), and the NHP (plain gray).

A quantitative analysis in terms of SSIM and CNR is presented in Figs. 9 and 10 for the three experiments.

The results are in agreement with what was observed visually and with the line profiles shown in Figs. 6 and 7. Standard and 1 mm (or 0.8 mm) static reconstructions exhibit lower SSIM than SR images, which showed a higher percentage of structural similarity in the order of 15–20% due to the oversampling introduced by the precisely corrected movement. Although not shown here, this quantitative analysis at different iterations yielded similar results.

Similarly, CNR results are in accordance with what was observed. Standard and 1 mm (or 0.8 mm) static reconstructions exhibit higher CNR in the order of 50% of increase due to the improved contrast recovery in small structures.

## 4. Discussion

This work shows that one can estimate PET images with a resolution that outperforms the intrinsic scanner resolution by harnessing, counter-intuitively perhaps, the usually undesired target motion, if measured at a higher resolution than the scanner’s intrinsic resolution. In other words, it is possible to not only compensate for the deleterious effects of motion on PET image quality, but to also lever-age the increased sampling information associated with moving targets to enhance the effective PET resolution based on super-resolution principles.

Although super-resolution has been investigated in PET before, to the best of our knowledge, this is the first study showing that it can be achieved with an external optical tracking device that is used to continuously measure head movement with a very high spatial and temporal precision during the listmode PET acquisition. The Polaris Vega tracking camera can indeed measure rigid-body transformations at a sampling rate of 60 Hz (~16.6 ms/frame) and with a much higher accuracy than the spatial resolution of the GE Discovery MI PET/CT scanner (0.12 mm vs. ~4 mm). Here, we exploited the measured subpixel motion in a listmode reconstruction framework with event-by-event repositioning which handles any type of movement in the 3D space, including unintentional motion, to achieve super-resolution.

An important step of the reconstruction is the generation of the sensitivity image. There are two main approaches to calculating the motion averaged sensitivity image (Rahmim et al., 2008). One approach consists in applying motion correction to the projection space followed by backprojection of all LORs, repeating this process for all poses ((Rahmim et al., 2008), Equation 16). Another strategy consists in performing only one backprojection and applying motion correction in the image space for all poses ((Rahmim et al., 2008), Equation 10). The first method can handle the attenuation correction properly but can be computationally expensive and very slow as the frame rate we are dealing with is very high (60 Hz). The second method is much faster but requires that the attenuation of the moving object is calculated during forward projection in the iteration process. Moreover, it requires segmenting the moving object from the static parts (scanner bed, static body etc.) in the attenuation map. Our results showed that the second approach, which was the one we used, works rather well. However, another disadvantage of this method is that by moving and averaging the sensitivity image, high frequency artifacts (in the form of Moiré patterns) gets propagated when a single ray Siddon projector (Siddon, 1985) is used for projection/backprojection and when the predefined voxel size is smaller than the scanner detector size. Using a multi ray Siddon projector (Moehrs et al., 2008) allowed to overcome this issue. The use of PSF modeling in the image space also contributed to alleviate most of the high frequency artifacts. However, multi ray Siddon algorithm is very computationally expensive, scaling with the number of rays used for each LOR. An alternative projector using a distance driven approach (Manjeshwar et al., 2006) is being studied to improve reconstruction time.

As previously described, we calibrated our system (Polaris tracker and PET/CT scanner) using paired position measurements in both respective spaces. Using CT image for spatial calibration offers arguably more accurate results than PET-based methods that use markers attached to a radioactive point source (Fulton et al., 2002) (super resolution would be difficult to achieve with such methods since the movement would be tracked at a resolution close to that of the PET scanner). However, determining the position of the center of a marker using CT images can be a limiting factor. Instead of comparing absolute positions of the object in the scanner and Polaris coordinate frames, we can compare the relative motion between two static positions in both spaces. This relative motion in the two systems is independent of any offset between the object position in the CT and Polaris coordinate frames. The advantage of this method is that it requires no careful measurement of the center of the marker sphere in the CT space, and, since a complex phantom can be measured, the relative position matrices for the CT data can be determined to a greater precision than the absolute position of the points in the former method.

Additionally, there are fundamental limits to super-resolution that should be considered. These limits are related to the spatial frequency content of the signal, which is constrained by the physical properties of the PET scanner. For instance, if the signal contains high-frequency components that are beyond the Nyquist limit of the system, they cannot be accurately captured even with super-resolution techniques. Overall, the diffraction limit and the finite number of detected events, impose a lower bound on the attainable resolution.

During all our experiments, the movements applied to the phantoms were mostly back and forth (translation and rotation). Hence, there was a predominant motion direction for each SR acquisition used in this work. It is possible that the applied motion patterns were not optimal to achieve oversampling and that the non-homogeneous nature of the movement implies a non-homogeneous resolution recovery. For instance, if the object is only rotating around a specific axis, the points belonging to that axis will not undergo motion and therefore will not benefit from the improvement offered by oversampling.

In a clinical context, the type and magnitude of motion can vary depending on the targeted population and disease. From our recent study (Tiss et al., 2022) on the impact of motion correction on longitudinal [^18^F]MK-6240 tau clinical brain scans, we found that 95% of the cohort of 65 subjects (55 Cognitively Normal, 7 with Mild Cognitive Impairment, and 3 with Alzheimer’s Disease) exhibited motion with an average displacement of 0.66 mm in X, 1.04 mm in Y, and 0.83 mm in Z axis. While this motion is within the accuracy range of the tracking setup, it’s at the lower bound of 0.7 mm, which is the limit imposed by the spatial calibration used in the study. To ensure isotropic enhancement of spatial resolution, it may be useful to impose motion on a patient’s head, for example, with a motorized massage pillow that provides continuous pseudo-random motion. Combined with the movement of the bed, this could provide sampling in all three directions.

**Fig. 11.**
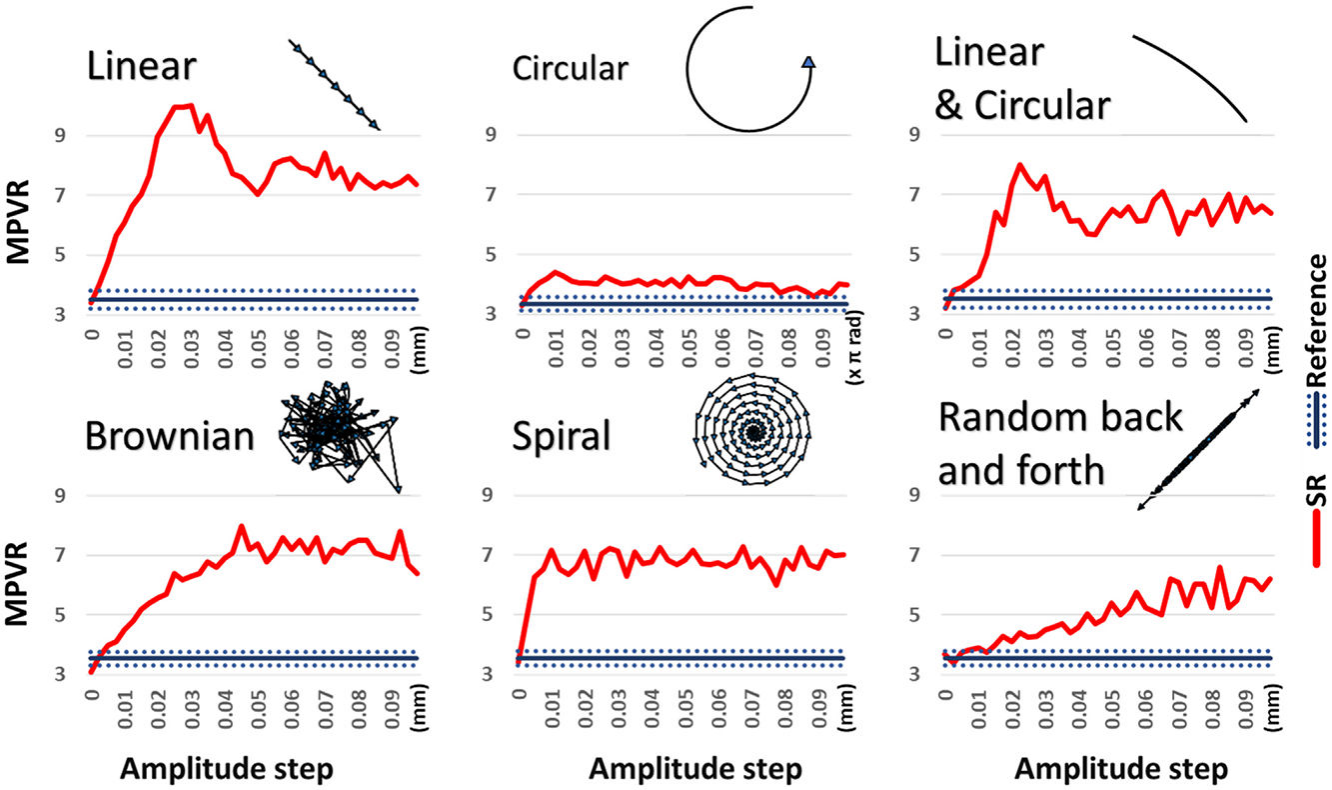
Illustration of the six patterns shapes we defined and plots of corresponding graphs displaying the measured resolution in terms of MPVR as a function of the amplitude step. The displayed patterns were obtained by applying the motion on a single point to have a sense of the path taken by the digital phantom for a given amplitude step. The MPVR were calculated on line profiles drawn on the reconstructed SR (red MPVR) and static reference images (blue MPVR). The dashed lines indicate one standard deviation around the mean reference MPVR.

To investigate the optimal motion patterns and amplitudes required for super-resolution, we conducted a simulation study using a high-resolution 2D phantom image consisting of nine hot spots of 2.4 mm in size. Six specific patterns of motion, including linear, circular, a combination of linear and circular (similar to that which was applied to our phantom experiments), Brownian-like, spiral, and random back and forth, were applied to the object on a hundred frames. The resulting list-mode data were then reconstructed using our super-resolution method and compared to a static reference. The mean peak-to-valley ratios (MPVR) of line profiles were calculated to quantify the benefits of super-resolution compared to the static reference. Our results showed that linear motion provided the highest super-resolution improvement, with a peak MPVR of 10 compared to an average of 3.5 for the static reference. Brownian-like motion, spiral motion and the combination of linear and circular motion also yielded significant super-resolution benefits, while circular motion and random back and forth motion resulted in less improvements Fig. 11.

These simulations showed that the specific motion amplitude required for optimal super-resolution benefits depends on the type of motion pattern used, but any type of pattern will lead to an increase in resolution as long as the amplitude is sufficient. We believe that any of these patterns or their combination will result in an increase in resolution in a clinical setup. However, these quantitative results depend on several parameters that we chose to fix for this study, and may not be generalizable to other scenarios. Overall, our findings provide valuable insights into the optimal motion patterns and amplitudes required for super-resolution, which can guide future clinical studies in this area.

It is possible to further improve super-resolved PET images quality by guiding the reconstruction using anatomical prior information (in a Bayesian sense). Such a regularization, allowing for a better noise control, could be important in SR as noise is exacerbated with smaller voxel size. Moreover, OSEM algorithm generally cannot be run to full convergence because the noise in the image grows with each iteration (Mettivier et al., 2011). To compensate for this, the algorithm is generally stopped after a determined number of iterations, resulting in an under-converged image. To address the effects of convergence and provide more accuracy in PET quantitation, a regularized reconstruction iterative algorithm will be studied, incorporating prior knowledge about the image into the reconstruction to better control noise propagation during SR reconstruction. This prior knowledge can be incorporated as segmented anatomical information from MR or CT images.

While we only tested this super-resolution technique in preclinical studies, we are currently working on extending it to human subjects. It is essential that no relative movement occurs between the markers and the head as those would decrease motion tracking accuracy and thus the performance of SR. In fact, we are focusing on ensuring that the relative motion of the markers attached to the object does not move beyond 0.7 mm. This level of accuracy is consistent with the spatial calibration method we use, which is critical for the impact of our super-resolution reconstruction. Moreover, the attachment device must be comfortable for long scans and must have minimal impact on CT and PET attenuation. Hence, we are working on developing a solution to rigidly attach markers to a subject’s head, consisting of a pair of swimming goggles to which a 3-D printed rod has been mounted to bring the Polaris markers to the top the head.

While the Polaris tracker can be effective in tracking the motion of an object’s surface, internal elastic deformations can be a limitation in clinical studies where we need to track the motion of internal organs. In our study, we focused on brain imaging, where internal deformations are negligible, and thus, we can apply rigid body transformations. However, this can be an important aspect if the proposed SR technique is to be extended to other clinical studies. In Marin et al. (2020) we have developed a motion correction for PET data using subspace-based real-time MR imaging in simultaneous PET/MR. By taking advantage of the high-resolution MR, we potentially will be able to generate deformation fields at a better resolution than the intrinsic PET spatial resolution, allowing for super-resolution reconstruction. We believe that such high-resolution non-rigid registration techniques, to track internal motion and adjust for the deformation in the image reconstruction process is a promising direction for future research.

One of the end goals of this work is to provide a methodology that will enable the detection of very early neurofibrillary tangles (NFTs) in regions. One of them is the locus coeruleus (LC), a small structure where tau pathology appears first in Alzheimer’s Disease, decades before symptoms. New data suggest that the LC is one of the earliest sites of tau pathology in AD and the initiator in the transmission of NFT during the progression of AD; however, its elongated shape and ultra-small dimensions, ~6–22 mm3 (Theofilas et al., 2017), pose a significant challenge for PET even with state-of-the-art scanners. Super-resolution may allow improved imaging of the LC.

## 5. Conclusion

We demonstrated that, in both phantom and animal studies, super-resolution can be achieved in brain PET by precisely measuring head movement in real time using a high-resolution infrared tracking camera. To attain this, we built a robust and accurate spatial and temporal calibration interface between a clinical scanner and the tracker. For both phantoms and NHP studies, the developed SR reconstruction method yielded PET images with visibly increased spatial resolution as compared to static acquisitions, allowing for improved visualization of small cortical and subcortical brain phantom structures. Improved PET resolution might allow for earlier and more accurate diagnosis of neurological disorders such as Alzheimer’s disease. It may also enable more accurate estimation of image-based input functions for quantification of dynamic brain PET studies.

## Data Availability

The data used in this work will be made available upon reasonable request, contingent on the obtention of a formal data sharing agreement with the institution(s) owning such data. However, the code in its current development is confidential. It will be made available in the future as part of an open-sourced reconstruction engine project for PET imaging or executable binary files will be provided upon reasonable request.
